# Concurrent disorders of cats with diabetes mellitus and arterial systolic hypertension

**DOI:** 10.1177/1098612X231187691

**Published:** 2023-07-20

**Authors:** Jonathon G Williams, Rebecka S Hess

**Affiliations:** Department of Clinical Sciences and Advanced Medicine, School of Veterinary Medicine, University of Pennsylvania, Philadelphia, PA, USA

**Keywords:** Blood pressure, amlodipine, diabetic, chronic kidney disease, hyperthyroidism

## Abstract

**Objectives:**

The aim of the present study was to report the concurrent disorders and treatment success of cats with diabetes mellitus (DM) and arterial systolic hypertension (SH).

**Methods:**

A retrospective longitudinal study was conducted of 17 cats with DM and SH that were examined at a university teaching hospital between 1 January 2011 and 31 December 2021. The medical records of diabetic cats were searched for the keywords ‘hypertension’, ‘blood pressure’, ‘amlodipine’, ‘benazepril’ and ‘telmisartan’ to identify cats with SH, which was defined as systemic arterial blood pressure (SABP) ⩾160 mmHg, documented at least twice, over several days. Comorbidities, including chronic kidney disease and hyperthyroidism, were recorded. Medications used for the treatment of SH and the SABP response to treatment were also noted.

**Results:**

Most cats (13/17, 76%) with DM and SH had at least one other documented concurrent illness that could contribute to SH, including chronic kidney disease (12/17 cats, 71%), hyperthyroidism (4/17, 23%) and functional adrenocortical mass secreting either aldosterone alone (1/17, 6%) or glucocorticoids, and possibly also aldosterone (1/17, 6%). Out of 17 cats, 15 (88%) were treated with amlodipine, and none were treated with an angiotensin converting enzyme inhibitor or an angiotensin II receptor blocker. Mean SABP at the time of diagnosis of SH was 210 ± 23 mmHg and was significantly higher than the mean SABP at the first and second follow-up examinations after the introduction of amlodipine treatment (175 ± 33 mmHg, *P* = 0.008 and 172 ± 26 mmHg, *P* = 0.01, respectively).

**Conclusions and relevance:**

Cats with DM and SH should be evaluated for the presence of chronic kidney disease, hyperthyroidism and functional adrenal masses. Treatment with amlodipine appears to be effective in lowering SABP in cats with DM and SH.

## Introduction

Systolic hypertension (SH) in cats with diabetes mellitus (DM) has been reported infrequently in a small number of small studies, and the concurrent disorders and clinical characteristics of this subgroup of cats with DM are not known.^1–4^ In one of these studies, which included 14 cats with DM and 19 healthy cats, SH was defined as a systemic arterial blood pressure (SABP) >180 mmHg, and SH was not documented in any of the study cats.^2^ However, in that study, mean SABP in the cats with DM was 161 ± 17 mmHg and median SABP was 167 mmHg, indicating that if a cutoff of 160 mmHg had been used to define SH, some cats with DM could have had SH.^1,2^ Cats were not evaluated for hyperthyroidism or chronic kidney disease (CKD) in the study.^2^ However, no proteinuria was noted in 12/14 diabetic cats in which it was measured, ocular examinations were normal in all 14 diabetic cats and echocardiographic findings were not significantly different from those observed in normal cats.^2^ Another study documented an SABP ⩾160 mmHg in 3/8 (37.5%) cats with DM.^3^ However, the aim of that study was to compare methodologies for SABP measurements, rather than describe concurrent disorders among cats with SH and DM.^3^ A third study evaluated 66 cats with DM, 35 non-diabetic cats with another illness and 11 healthy cats.^4^ In the study, SH was defined as an SABP ⩾160 mmHg and 15% of cats with DM were determined to have SH.^4^ Cats with azotemia and hyperthyroidism were excluded from the study but the urine protein:creatinine ratio was found to be significantly higher in diabetic cats (0.81) compared with healthy cats (0.24, *P* <0.001).^4^ All three of these studies used Doppler ultrasonography to determine SABP, and none reported the response to antihypertensive treatment.^2^–^4^

Untreated hypertension in cats is associated with proteinuria, glomerular disease, retinopathy, encephalopathy and cardiomyopathy.^1^ Improving the understanding of the clinical characteristics and concurrent disorders observed in cats with SH and DM could influence the timing of diagnostic efforts targeted at investigating the presence of SH in cats with DM. Advancing the knowledge of the clinical characteristics and comorbidities of cats with DM and SH could also improve the therapeutic interventions and outcomes of these cats. Furthering the understanding of the clinical characteristics and comorbidities of cats with DM and SH can also help inform veterinarians and owners about the long-term prognosis, quality of life and expected response to treatment of this subgroup of diabetic cats. The aims of this study were therefore to determine the clinical characteristics, comorbidities and SABP response to antihypertensive treatment in cats with DM and SH.

## Materials and methods

### Case selection

For this retrospective longitudinal study, an electronic search of medical records identified all cats with DM examined at a university teaching hospital between 1 January 2011 and 31 December 2021. Records were searched for the keywords ‘diabetes mellitus’, ‘diabetic’ and ‘diabetes’ to identify cats with DM. The search also determined if any of the keywords ‘hypertension’, ‘blood pressure’, ‘amlodipine’, ‘benazepril’ and ‘telmisartan’ were identified in the records of cats with DM. Cats in which keywords suggesting DM and SH were identified, and were defined as cats with a suspicion of DM and SH. The medical records of all cats with a suspicion of DM and SH were reviewed in detail by one person (JGW) to confirm that the cats did indeed have DM and SH. Cats were defined as diabetic if they were insulin-treated at the time SH was diagnosed. Cats in diabetic remission at the time SH was diagnosed were excluded from the study. A diagnosis of SH was confirmed if at least two SABP measurements documented elsewhere or at the university teaching hospital were ⩾160 mmHg. Cats with repeated SABP measurements ⩾160 mmHg were included in the group of cats with confirmed DM and SH, even if the measurements were performed over a period of several days during the same inpatient hospital visit. Cats with a suspicion of SH were excluded from the group of cats with DM and SH if they had been previously treated for SH but treatment was discontinued and there was no documented record of a current SABP ⩾160 mmHg.

### Medical records review

The medical records of cats with DM and a suspicion of SH were reviewed in detail, and the following data were recorded: sex; breed; age; body weight; body condition score (1–9); ocular examination findings; age at the time of DM and SH diagnoses; insulin type, dose and frequency of administration; urine specific gravity; urine protein:creatinine ratio; and serum creatinine, symmetric dimethylarginine and total thyroxine concentrations.^5^ Blood pressure measurements at the time of diagnosis of SH, as well as at the time of follow-up examinations, were recorded, as was the type, dose and frequency of administered antihypertensive medication, and the time that elapsed between SABP measurements at follow-up examinations. The highest SABP recorded for each cat at each visit was reported. Doppler ultrasonography is the standard-of-care method for indirect arterial SABP measurements in cats at this hospital; however, the method of acquisition was not noted for all samples (Ultrasonic Doppler Flow Detector; Parks Medical Electronics). Comorbidities at the time of SH diagnosis were also recorded. CKD was diagnosed and classified based on the International Renal Interest Society guidelines.^6^ Hyperthyroidism was diagnosed if the cat had been previously diagnosed with hyperthyroidism and was being treated for hyperthyroidism at the time of examination or if the total thyroxine concentration was greater than 3.8 μg/dl at the time of diagnosis of SH.^7^ The results of echocardiograms and abdominal ultrasonograms interpreted by an American College of Veterinary Internal Medicine (Cardiology) and an American College of Veterinary Radiology boarded clinician, respectively, were also reported.

### Statistical analysis

Counts and percentages are reported for categorical variables. Distribution of continuous variables was assessed for normality by visual inspection of a histogram and by the skewness and kurtosis tests.^8^ Continuous variables with a normal distribution are reported as mean and standard deviation, whereas continuous variables that are not normally distributed are reported as median and range. The paired *t*-test was used to compare normally distributed continuous variables, whereas the Wilcoxon signed rank sum test was used for comparisons of continuous variables that were not normally distributed. *P* <0.05 was considered significant for all tests. All statistical evaluations were performed using a statistical software package (Stata 14.0 for Mac; Stata Corp).

### Results

A total of 32 cats with DM had a suspicion of SH, but in 12 cats, the suspicion for SH was not confirmed. These 12 cats comprised 10 cats that had only one documented measurement of SH and two cats in which previous treatment for SH had been discontinued and SABP was normal at the time of examination. Three additional cats were excluded because they were in diabetic remission at the time SH was documented. Ultimately, the study included 17 cats with DM and SH. During the same period in which the cats with DM and SH were examined, 733 other cats with DM and no documented SH were examined at the hospital, though it is unclear how many of these cats had SABP measured.

The data for cats with DM and SH are reported in 17 cats, unless specifically noted. The mean age at the time of SH diagnosis was 14.7 ± 2.7 years; 16 (94%) cats were domestic shorthair cats, one (6%) was a Bengal cat, nine (53%) were spayed female cats and eight (47%) were castrated male cats. The mean weight was 4.5 ± 1 kg and the median body condition score reported in only 7/17 (41%) cats was 6 (range 3–7). A complete ocular examination was documented in 4/17 (23%) cats. Retinal detachment was identified in one of these cats, a healed corneal ulcer in the right eye was identified in another and an unspecified lesion in the fundus of the left eye was identified in the third cat. The fourth cat had negative menace, positive dazzle, incomplete pupillary light reflex, hyper-reflective tapetum and pinpoint retinal hemorrhages in both eyes.

The mean age of cats with DM and SH at the time of DM diagnosis was 12.1 ± 2.8 years and the mean duration of insulin treatment before diagnosis of SH was 2.4 ± 3 years. The type of insulin administered to cats with DM and SH was glargine (12/17, 70%), porcine lente (2/17, 12%), protamine zinc recombinant human insulin (1/17, 6%) or unknown (2/17, 12%). Most cats received insulin twice daily (14/17, 82%) and the remainder received insulin once daily (2/17, 12%) or insulin at an unknown frequency of administration (1/17, 6%). The median insulin dose administered to cats with DM and SH was 0.2 U/kg/dose (range 0.1–0.8 U/kg/dose).

SABPs were recorded from the time of diagnosis of SH and at the next two follow-up examinations, if available. All reported that SABP measurements were obtained at the university teaching hospital. The mean SABP at the time of diagnosis of SH and during the next two follow-up examinations, performed for treatment monitoring, were 210 ± 23 mmHg, 175 ± 33 mmHg and 172 ± 26 mmHg, documented in 17/17 (100%), 16/17 (94%) and 10/17 (59%) cats, respectively. The mean time between measurement of SABP at the time of diagnosis of SH and the first follow-up examination was 9.7 ± 11.2 weeks, and the mean time between the first and second follow-up examinations was 8.4 ± 9.9 weeks. The mean SABP at the time of diagnosis of SH was significantly higher than at the time of the first and second follow-up examinations (*P* = 0.008 and *P* = 0.01, respectively), but the mean SABP at the first follow-up examination was not significantly different from the mean SABP at the second follow-up examination.

Of the 17 cats, 15 (88%) were treated with amlodipine, two were treated with an unknown medication and none had documented treatment with an angiotensin converting enzyme inhibitor or an angiotensin II receptor blocker. The median amlodipine doses administered at the time of diagnosis of SH and during the next two follow-up examinations were 0.149 mg/kg/day (range 0.107–0.355), 0.147 mg/kg/day (range 0.107–0.49) and 0.131 mg/kg/day (range 0.118–0.281), reported in 15/17 (88%), 13/17 (76%) and 9/17 (53%) cats, respectively. The median amlodipine dose administered at the time of diagnosis of SH was significantly higher than at the time of the first and second follow-up examinations (*P* = 0.01 and *P* = 0.008, respectively), and the median amlodipine dose administered at the first follow-up examination was also significantly higher than the median amlodipine dose administered at the second follow-up examination (*P* = 0.01). At the time amlodipine treatment started, it was given once daily in 10/15 (67%) cats and twice daily in 5/15 (33%) cats. At the time of the first follow-up examination, amlodipine was given once daily in 8/13 (62%) cats and twice daily in 5/13 (38%) cats. At the time of the second follow-up examination, amlodipine was given once daily in 3/9 (33%) cats and twice daily in 6/9 (67%) cats.

Most cats (13/17, 76%) with DM and SH had at least one other concurrent illness, including CKD (diagnosed in 12/17 cats, 71%) and hyperthyroidism (diagnosed in 4/17 cats, 23%). The mean creatinine concentration at the time of diagnosis of SH in the 12 cats with CKD was 2.5 ± 0.8 mg/dl, and 8/12 cats (67%) were classified as stage 2, while 4/12 cats (33%) were classified as stage 3, according to the International Renal Interest Society guidelines. The median urine specific gravity in 12/17 (71%) cats with DM and SH was 1.016 (range 1.011–1.101) and 10/12 (83%) of these cats also had CKD. Of the 12 cats with DM and SH that had documented urine specific gravity, three (25%) had both CKD and hyperthyroidism. The median total thyroxine concentration in the four cats with hyperthyroidism at the time of diagnosis of SH was 5 μg/dl (range 4.2–9.1). Nine other cats had normal total thyroxine concentration, and one other cat was diagnosed with hyperthyroidism 10 months after the time of diagnosis of SH, with a total thyroxine concentration of 4.2 μg/dl. All but one of the cats with CKD had a total thyroxine concentration measured. None of the cats with DM and SH had symmetric dimethylarginine concentration measured, and only 2/17 cats with DM and SH had urine protein:creatinine ratio measurements reported as 5.01 and 0.14, respectively. Of the 17 cats, 10 (59%) had the results of an echocardiogram available for review. Left ventricular remodeling with areas of thickening was the cardiac diagnosis in five of these 10 cats (50%), and was interpreted as consistent with age-related changes, changes secondary to systemic disease (such as hyperthyroidism or hypertension) or possible early primary cardiac disease, such as hypertrophic cardiomyopathy. No specific cardiac therapy was recommended in these five cats. Of the 10 cats, three (30%) had concentric left ventricular hypertrophy consistent with hypertrophic cardiomyopathy, hyperthyroidism or systemic hypertension, and 2/3 cats had congestive heart failure, which required specific cardiac therapy with oxygen, furosemide, benazepril, clopidogrel and pimobendan. The third cat required no specific cardiac therapy. Of these 10 cats, one (10%) had four-chamber dilation consistent with volume overload, which required no specific cardiac therapy, and one (10%) had a normal cardiac examination.

The results of an abdominal ultrasonography were available in 16/17 (94%) cats. The most commonly observed abdominal ultrasound abnormalities included decreased renal corticomedullary definition consistent with chronic renal changes (noted in 14/16 cats, 87%), and of these 14 cats, three (21%) also had pyelectasia suggestive of acute on chronic kidney disease, heteroechoic pancreatomegaly consistent with chronic pancreatitis or pancreatic nodular hyperplasia (11/16, 69%) and hyperechoic hepatomegaly consistent with DM (10/16, 62%). Of the 16 cats, two (12%) had an adrenal mass. Hyperaldosteronism was suspected in both cats based on hypokalemia and imaging findings and was confirmed in one with an aldosterone concentration of 4522 pmol/l (reference interval 194–388). The other cat in which aldosterone concentrations were not measured had a low-dose dexamethasone suppression test consistent with a diagnosis of hyperadrenocorticism. Cortisol concentrations were 2.1 mcg/dl, 14.6 mcg/dl and 17.2 mcg/dl at 0, 4 and 8 h after intravenous (IV) administration of 0.1 mg/kg of dexamethasone. The cat underwent an adrenalectomy in which an adrenocortical carcinoma was confirmed. This cat also had congestive heart failure, and treatment with amlodipine was eventually changed to treatment with benazepril. Both cats with adrenal tumors also had CKD, but no documented hyperthyroidism.

## Discussion

This is the first study to report the concurrent disorders and treatment success of cats with DM and SH. In this study, which found that most cats with DM and SH had CKD or hyperthyroidism, none of the cats had mild, stage 1 CKD, according to the International Renal Interest Society guidelines, and median total thyroxine concentration in the cats with hyperthyroidism was 5 μg/dl, indicating that hyperthyroidism was also not mild.^7^ These results could suggest that cats with DM and SH should be screened for CKD and hyperthyroidism earlier than was the practice in this study. CKD and hyperthyroidism were also the most common concurrent disorders reported in a general population of 282 cats with SH.^9^ Therefore, it is possible that in cats, CKD and hyperthyroidism are more important contributors to SH than DM alone. In humans, myocardial infarct, stroke and heart failure are significantly more common in patients with DM and SH, compared with those with DM alone.^10,11^ Interestingly, DM does not appear to be a risk for arterial thromboembolism or cardiovascular disease in cats, further corroborating the notion that SH is uncommon in cats with DM alone.^12–16^

The findings of this study also suggest that amlodipine treatment significantly decreases SABP in cats with DM and SH, and that in most cats, the dose of amlodipine can be decreased at the time of follow-up examinations. These findings support the use of amlodipine to decrease SABP in cats with DM and SH. However, these findings are based on follow-up SABP measurements documented in only 16/17 (94%) and 10/17 (59%) study cats during the two follow-up visits, over a period of about 4.5 months. Urine protein:creatinine ratios were reported in too few cats for meaningful data interpretation in the current study, but a previous study of cats with DM found that proteinuria defined as a protein:creatinine ratio >0.4 was significantly more common in cats with DM compared with other cats, suggesting that in some cats, treatment with angiotensin converting enzyme inhibitors or angiotensin II receptor blockers could be beneficial.^4^ Future studies examining the use of different antihypertensive medications in cats with DM and SH, focused on cats with and without proteinuria, are indicated to determine the best treatment modalities for cats with DM and SH.

This study cannot report the hospital period prevalence of SH in cats with DM, because it is not definitively known that 733 diabetic cats examined during the study period with none of the keywords used to identify a suspicion for SH were normotensive. However, a confirmed diagnosis of SH in cats with DM appears rare in this study population. The decision not to have an SABP measurement performed in most cats with DM was likely based on a number of factors, including lack of clinical suspicion for hypertension, owner preference and cat temperament. Future prospective studies in which all cats with DM have an SABP measurement, regardless of a clinical suspicion for SH, are needed in order to determine the prevalence of SH in cats with DM. Future studies could also compare the prevalence of SH, treatment success of SH and concurrent illness in cats with SH, with and without DM.

The results of this study suggest that a diagnosis of SH is uncommon among cats with DM and this finding is consistent with another larger study of 282 cats with SH, in which only 2% of cats had DM.^9^ The uncommon occurrence of SH in cats with DM is different from what is observed in dogs and humans with DM. In dogs with DM, the reported prevalence of SH is in the range of 46–67%, but these estimates may be inaccurate because they are based on small studies that include 14–50 dogs with DM.^17–19^ In humans, the Framingham study estimated that 58% of 1145 newly diagnosed diabetic individuals who did not have a previous history of cardiovascular events had hypertension at the time of the DM diagnosis.^10^ This finding is clinically important because in humans, hypertension increases the risk of all-cause death by 72% and the risk of any cardiovascular event in those with DM by 57%.^10^ The pathophysiology of SH in DM is multifactorial and incompletely understood. Diabetic nephropathy, obesity, arterial stiffness, inappropriate activation of the renin–angiotensin–aldosterone system and sympathetic nervous system, mitochondrial dysfunction and oxidative stress leading to increased intracellular reactive oxygen species that worsen arterial stiffness, and proinflammatory systemic and cardiovascular cytokines have all been implicated in the pathophysiology of SH in DM in humans.^11^ Currently there are no data to suggest that DM is a risk for SH in cats. Although one study reported that the mean SABP in 66 diabetic cats (144 ± 24 mmHg) was significantly higher than the mean SABP in 11 healthy controls (134 ± 21 mmHg), the diabetic cats in this study also had a significantly higher urine protein:creatinine ratio compared with healthy controls; serum creatinine and blood urea concentration were not reported, so it is not known if the difference in SABP was due to renal disease or DM.^4^ An improved understanding of species differences as they relate to cardiovascular disease, and further research into why cats with spontaneous DM develop SH less frequently compared with dogs and humans with DM, could help elucidate the mechanisms of SH in DM further.

This study has several limitations associated with the retrospective study design. First, the measurement of SH was decided based on the clinical judgment of a large number of clinicians and the decision to measure SABP could have been influenced by the presence of concurrent illness, such as CKD and hyperthyroidism. This could have skewed findings to diagnose SH in cats with DM and comorbidities more commonly than in cats with DM and no comorbidities. This limitation is consistent with another retrospective study of SH in cats, in which only 1.34% of 347,889 cats had an SABP measurement.^9^ In addition, not all cats had an echocardiogram performed, and most cats did not have a complete documented ocular examination; therefore, it is possible that the prevalence of cardiac and ocular abnormalities in cats with DM and SH is underestimated in this study. It is possible that some cats had an ocular examination that was not documented in the medical record. Another study limitation is that Doppler ultrasonography rather than direct SABP measurements are reported. Although Doppler ultrasonography has been reported to be more accurate than oscillometric devices in cats with DM, it is possible that the reported measurements are inaccurate.^3^ Furthermore, because the standard-of-care method for indirect arterial SABP measurements in cats at this hospital is Doppler ultrasonography, this was not definitively stated in the record. Finally, the mean time between follow-up SABP measurements was approximately 8–10 weeks, which is longer than the 4–8 weeks recommended for cats with an SABP in the range of 160–179 mmHg, or the 1–2 weeks recommended for cats with an SABP ⩾180 mmHg.^1^ The retrospective study design did not allow for standardization of the timing of follow-up examinations, dosing of medications and diagnostic tests performed.

## Conclusions

It is concluded that SH in cats with DM is observed mainly in cats with CKD and hyperthyroidism. Adrenal tumors were also diagnosed in a small number of cats with DM and SH. It is therefore recommended that cats with DM and SH be evaluated for the presence of these concurrent illnesses. In addition, treatment with amlodipine is effective in reducing SABP in cats with DM and SH.
